# Persistent Posttetanic Depression at Cerebellar Parallel Fiber to Purkinje Cell Synapses

**DOI:** 10.1371/journal.pone.0070277

**Published:** 2013-07-29

**Authors:** Astrid Bergerot, Mark Rigby, Guy Bouvier, Païkan Marcaggi

**Affiliations:** 1 Department of Neuroscience, Physiology and Pharmacology, University College London, London, United Kingdom; 2 Institut de Biologie de l’Ecole Normale Supérieure, Paris, France; 3 INSERM U1024, Paris, France; 4 CNRS UMR8197, Paris, France; Centre national de la recherche scientifique, University of Bordeaux, France

## Abstract

Plasticity at the cerebellar parallel fiber to Purkinje cell synapse may underlie information processing and motor learning. *In vivo*, parallel fibers appear to fire in short high frequency bursts likely to activate sparsely distributed synapses over the Purkinje cell dendritic tree. Here, we report that short parallel fiber tetanic stimulation evokes a ∼7–15% depression which develops over 2 min and lasts for at least 20 min. In contrast to the concomitantly evoked short-term endocannabinoid-mediated depression, this persistent posttetanic depression (PTD) does not exhibit a dependency on the spatial pattern of synapse activation and is not caused by any detectable change in presynaptic calcium signaling. This persistent PTD is however associated with increased paired-pulse facilitation and coefficient of variation of synaptic responses, suggesting that its expression is presynaptic. The chelation of postsynaptic calcium prevents its induction, suggesting that post- to presynaptic (retrograde) signaling is required. We rule out endocannabinoid signaling since the inhibition of type 1 cannabinoid receptors, monoacylglycerol lipase or vanilloid receptor 1, or incubation with anandamide had no detectable effect. The persistent PTD is maximal in pre-adolescent mice, abolished by adrenergic and dopaminergic receptors block, but unaffected by adrenergic and dopaminergic agonists. Our data unveils a novel form of plasticity at parallel fiber synapses: a persistent PTD induced by physiologically relevant input patterns, age-dependent, and strongly modulated by the monoaminergic system. We further provide evidence supporting that the plasticity mechanism involves retrograde signaling and presynaptic diacylglycerol.

## Introduction

The cerebellum is essential for fine tuning of motor coordination and motor learning [1], [2]. Its cortex is made of neuronal microcircuits whose strikingly repetitive architecture has led to speculations regarding their computational design, pointing at the granule cell (GC) to Purkinje cell (PC) excitatory synapse as the main locus for long-term synaptic plasticity [3]. This synapse has since attracted attention from physiologists and was shown to exhibit long-term depression (LTD), first reported by Matsuo Ito, three decades ago [4].

Following Ito’s discovery, the cellular and molecular mechanisms involved in LTD have been intensively investigated. Although its induction mechanism has remained controversial [5], [6], [7], [8], it is generally accepted that it requires a large postsynaptic calcium rise and that its expression is mediated by postsynaptic AMPA receptor phosphorylation and internalization [6], [9], [10]. This postsynaptic LTD has later been shown to be reversible: a postsynaptic long-term potentiation (LTP) can be evoked by protocols producing smaller postsynaptic calcium rises [11], [12]. The picture is less complete for plasticity expressed as a change in the amount of neurotransmitter release (presynaptic plasticity). Sustained 4–8 Hz firing of parallel fibers (PFs; GC axons) has been reported to induce either a protein kinase A (PKA)-mediated presynaptic LTP [13], or a presynaptic LTD when PKA is blocked [14], albeit the physiological occurrence of the latter, in the absence of inhibitors, remains unknown.

All the protocols used to induce long-term plasticity at the GC to PC synapse have involved repetitive firing at low frequency. There is no evidence that such input patterns occur *in vivo*, where GCs have been reported to fire in short bursts at high frequency [15]. A single burst of high frequency PF firing evoked by tetanic stimulation is known to induce endocannabinoid-mediated short-term depression [7], [16], [17], but only for spatially dense input patterns [17], [18], the occurrence of which is unclear *in vivo*. Here we report that tetanic PF stimulation also evokes a longer lasting depression, which does not depend on the spatial pattern of the input as the endocannabinoid-mediated plasticity does. This persistent posttetanic depression (PTD) lasts at least 20 min, however, due to its small amplitude (7–14%), we have not attempted to quantify its duration beyond this time point. In contrast to the classic postsynaptic LTD, the persistent PTD is associated with an increase in paired-pulse facilitation and coefficient of variation of synaptic responses, consistent with a presynaptic expression. We show that the induction requires postsynaptic calcium, suggesting mediation by a retrograde signal. Extensive pharmacological investigation ruled out endocannabinoids and other putative retrograde messengers. Finally, we show that this novel form of plasticity is permitted by tonic levels of monoamines.

## Materials and Methods

### Ethics Statement

In accordance with the United Kingdom Animal (Scientific Procedures) Act of 1986, this study was covered by the Home Office project license PPL 70/05771 for the breeding GCaMP2 (transgenic) mice. Mice were humanely killed at a designated establishment by cervical dislocation, which is an appropriate method under Schedule 1 of the Act.

### Cerebellar Slice Preparation

We used C57Black/6 wild type or ICR transgenic mice expressing the fluorescent calcium indicator protein GCaMP2 under the control of regulatory sequences of the gene coding Kv3.1 potassium channel which, in the cerebellum, is expressed only in GCs [19]. The latter mice were referred to as GCaMP2 mice. 24 to 40 day old mice were used unless otherwise stated. They were humanely killed by cervical dislocation before being decapitated (see Ethics statement). The brain was swiftly extracted and immersed in ice cold incubation medium. The cerebellum was dissected and glued on agar with tissue adhesive (3 M Vetbond). Vermis sagittal slices 250 µm thick were cut in slicing medium with a VT1200S slicer (Leica). Slices were then incubated at 35°C for 20 min and then at room temperature for up to 8 hours before recording.

### Slice Superfusion and Electrophysiology

Recordings were obtained from slices held in a 1 ml bath on the stage of an Olympus BX51 microscope, and superfused at 2 ml min^−1^. Experiments were performed at room temperature unless otherwise stated. Recording and stimulating pipettes were positioned at 75–100 µm distance on the z-axis, so one of the pipettes had to be placed deep in the slice. The Purkinje cells were whole-cell voltage-clamped to −65 mV under visual control, series resistance was typically 5 MΩ and not compensated. Experiments were analyzed only when series resistance drift was negligible. Extracellular recordings were made in current clamp with 1 µm tip pipettes filled with extracellular solution. Patch-clamp and extracellular recordings were made using a Multiclamp 700 B (Molecular Devices). Stimulations were typically negative square 10 V × 100 µs pulses applied through a 1.5 MΩ glass electrodes.

### Solutions

**Figure 1:**
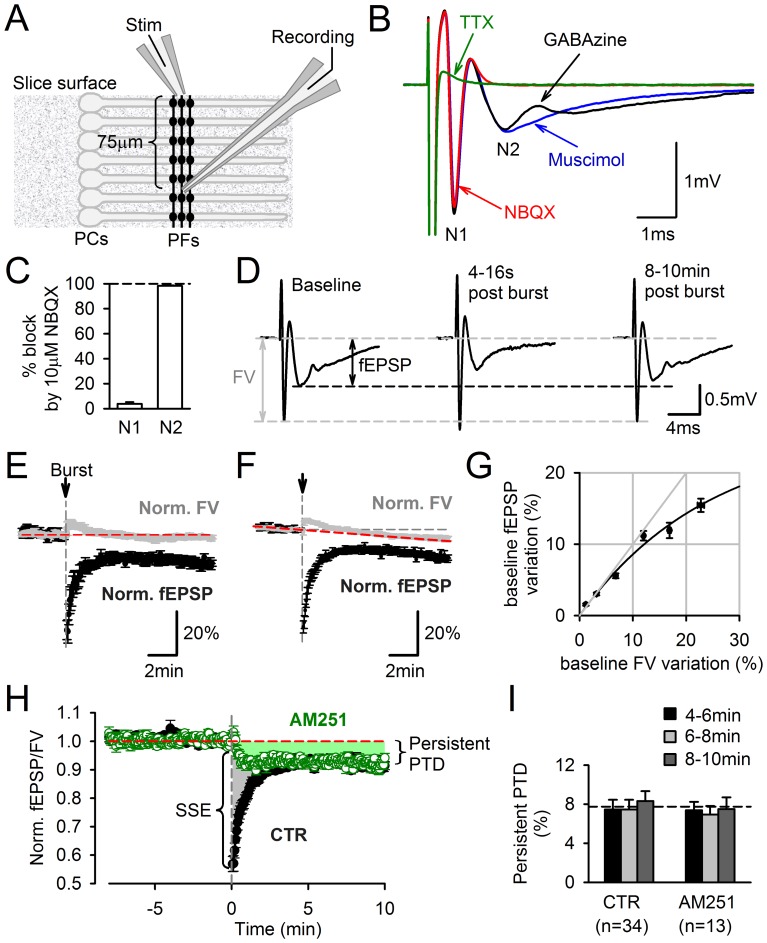
Persistent PTD evoked by a single burst of PF firing. **A**, Schematic diagram of electrode positions in a sagittal cerebellar slice for field potential recording of the PF to PC synaptic transmission. The effect of PF stimulation was recorded extracellularly at least 70 µm vertically below the stimulation electrode. **B**, Typical field potential evoked by a single PF stimulation. In 10 µM GABAzine (black trace), three negative peaks were resolved. The second negative peak (N2) was abolished in 25 µM NBQX (red trace) and unaltered by the GAGA_A_ agonist muscimol (10 µM). The N2 peak was assimilated to the field excitatory postsynaptic response (fEPSP). The first negative peak (N1), which remained in NBQX and was abolished in 1 µM TTX, was assimilated to the fiber volley (FV), i.e. the field potential associated to the action potential propagation along PFs. **C**, The N1 and N2 peaks are respectively 96.2±1.4% and 1.7±0.3% of control in 10 µM NBQX (n = 9). **D**, Effect of a burst of 10 stimuli at 200 Hz on the recorded field potential. Traces from left to right are averaged field potentials evoked by 15 consecutive stimulations for the 2 min before the burst, 4 consecutive stimulations starting 4 s after the burst, and 15 consecutive stimulations starting 8 min after the burst. A depression of the fEPSP is observed 8–10 min after the burst stimulation. This depression was named persistent PTD (see Text). **E**, Time course of FV (grey) and fEPSP (black) amplitudes normalized to their averaged values for the 2 min preceding the burst. Only recording showing no detectable change in FV amplitude over their duration are plotted (n = 13). **F**, Same as in E, but including all the recordings exhibiting an averaged change in the FV amplitude of less than 1%/min (n = 34). Although on average the FV decreased by 5.9±0.8% after 10 min, the fEPSP depression was significantly larger (14±0.8%, n = 34, p = 3·10^−9^). **G**, Average change in fEPSP amplitude plotted against change in FV amplitude. Data obtained from baseline recordings preceding burst stimulation (n = 34). The relationship between FV and fEPSP is sublinear rather than supralinear. **H**, The persistent PTD is not CB1 receptor-dependent. Synaptic transmission is plotted as fEPSP/FV normalized to the averaged value during the two minutes preceding the burst stimulation. In control condition (n = 34), the burst evokes an SSE (transient 40% depression, see Text) and a persistent PTD (remaining 8% depression until at least 10 min after burst). Following incubation with 2 µM AM251, the SSE (shaded grey) was abolished while the persistent PTD (shaded green) remained unchanged (n = 13). **I**, Average persistent PTD amplitude 4–6 min (black), 6–8 min (grey) and 8–10 min (dark grey) following the burst, in control and AM251, showing that steady amplitude is reached after 4 min.

**Figure 2:**
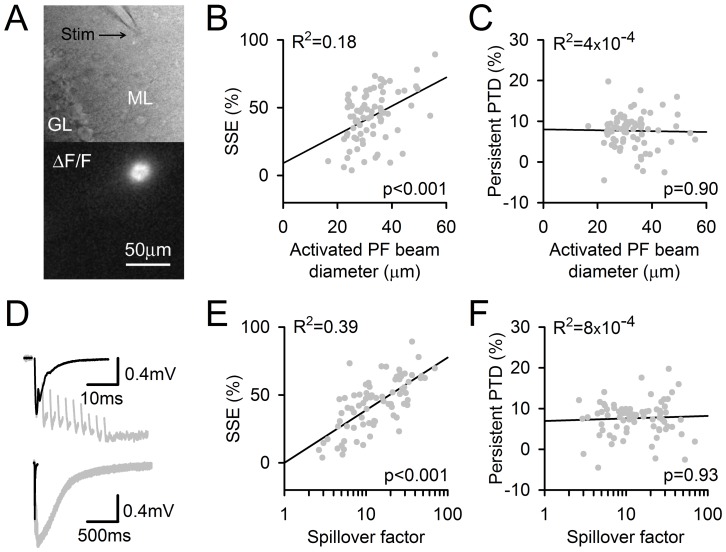
The SSE and the persistent PTD are differentially affected by the input pattern and the amount of glutamate spillover. ***A,*** Image of a beam of activated PFs in a sagittal slice. Top, position of the stimulating electrode in the molecular layer (ML). Bottom, ΔF/F signal arising from GCaMP2 in PFs, following their stimulation. The beam of stimulated PFs was assimilated to a cylinder, the diameter of which was calculated (see Methods). ***B,*** The SSE amplitude correlates with the PF beam diameter (p = 0.0004). ***C,*** The persistent PTD amplitude does not correlate with the PF beam diameter (p = 0.90). ***D,*** Specimen traces illustrating the spillover effects on postsynaptic responses. The fEPSP evoked by one pulse (black line) is superimposed to the fEPSP evoked by a 10-pulse 200 Hz train (grey; FV has been erased for clarity). The two fEPSPs are shown on a longer time scale to show the striking difference in their decay time course (bottom). The prolongation of this decay was used to calculate the spillover factor (see Text; (Marcaggi *et al.*, 2003)). ***E,*** The SSE amplitude correlates to the spillover factor (p<2·10^−7^). ***F,*** In striking contrast, the persistent PTD does not correlate with the spillover factor (p = 0.93). R^2^, the square of the correlation coefficient between the data and their linear regression (black lines) is indicated for each plot. Data were collected from 76 recordings obtained for monitoring frequencies between 0.0625 and 0.25 Hz and lasting for at least 6 min following the 10-pulse burst at 200 Hz.

**Figure 3:**
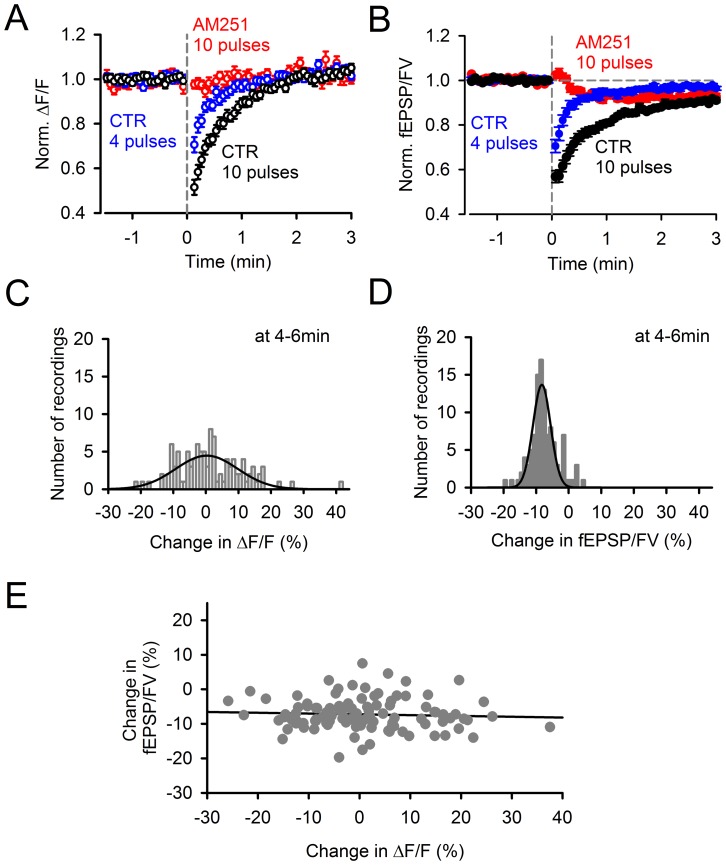
The persistent PTD is not associated with detectable changes in presynaptic calcium signaling. ***A,*** Effect of a 200 Hz burst on the PF action potential-evoked residual presynaptic calcium transients. Presynaptic calcium transients evoked by double pulse stimulation were recorded as the normalized change in fluorescence (ΔF/F) of GCaMP2 expressed in the axons of granule cells. ΔF/F was reduced during the minute following induction by a train of 10 (n = 34; black) or 4 (n = 35; blue) pulses at 200 Hz. However, no reduction of ΔF/F was observed in 2 µM AM251 (n = 13; red). ***B,*** Normalized fEPSP/FV for the same data, on the same expanded time scale for comparison with (**A**). ***C,*** Distribution histogram of the change in ΔF/F 4–6 min after a 10-pulse burst at 200 Hz (n = 107; pooled control and conditions in which neither the persistent PTD nor synaptic transmission were affected). This distribution exhibits a high variability partly due to the low signal-to-noise of the ΔF/F recordings. Gaussian fit peaks at +0.3%. ***D,*** Distribution histogram of the change in fEPSP/FV 4–6 min following induction (i.e., the opposite of persistent PTD amplitude) for the same recordings. Gaussian fit peaks at −8.3%. ***E,*** Data shown in (***D***) plotted against data shown in (***C***). The persistent PTD amplitude does not correlate with ΔF/F (p = 0.6; Spearman Rank correlation).

**Figure 4:**
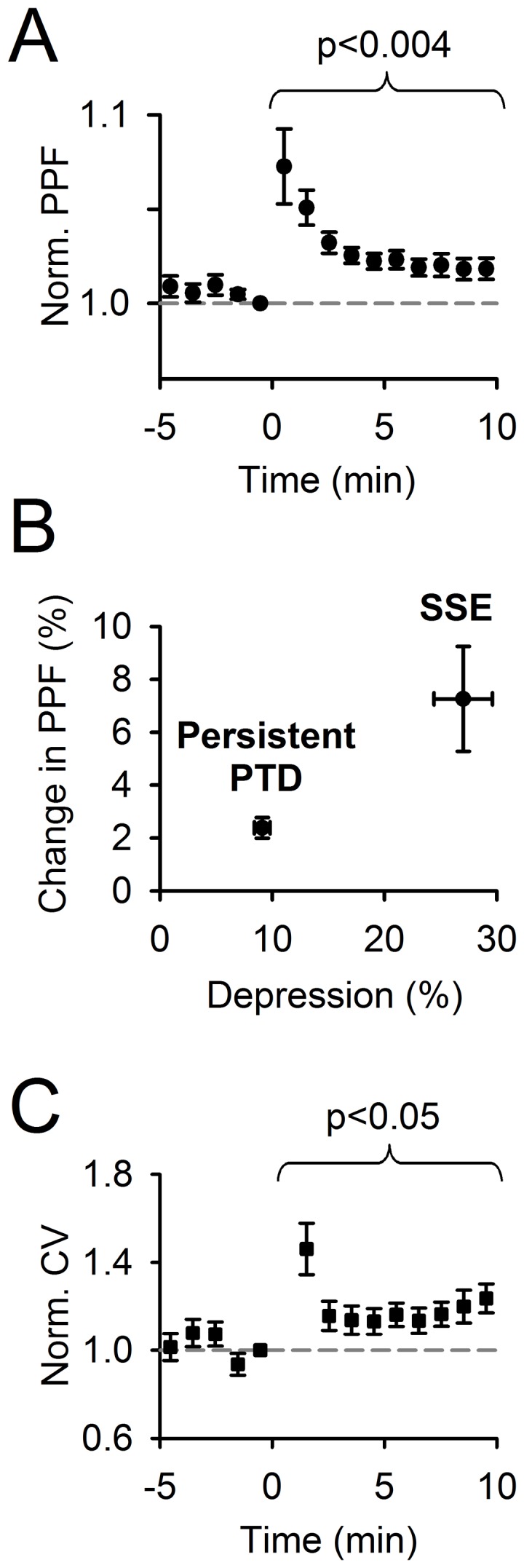
Data supporting presynaptic expression of the persistent PTD. ***A,*** Averaged change in the paired-pulse facilitation (PPF) of fEPSP/FV (n = 27). ***B,*** Averaged change in the PPF 0–1 min (SSE) and 8–10 min (persistent PTD) following induction plotted against averaged percentage depression over the same time ranges. ***C,*** Averaged change in coefficient of variation (CV) for the same data set. Actual averaged CV before the induction (at time point −1 min) was 3.0±0.4%.

**Figure 5:**
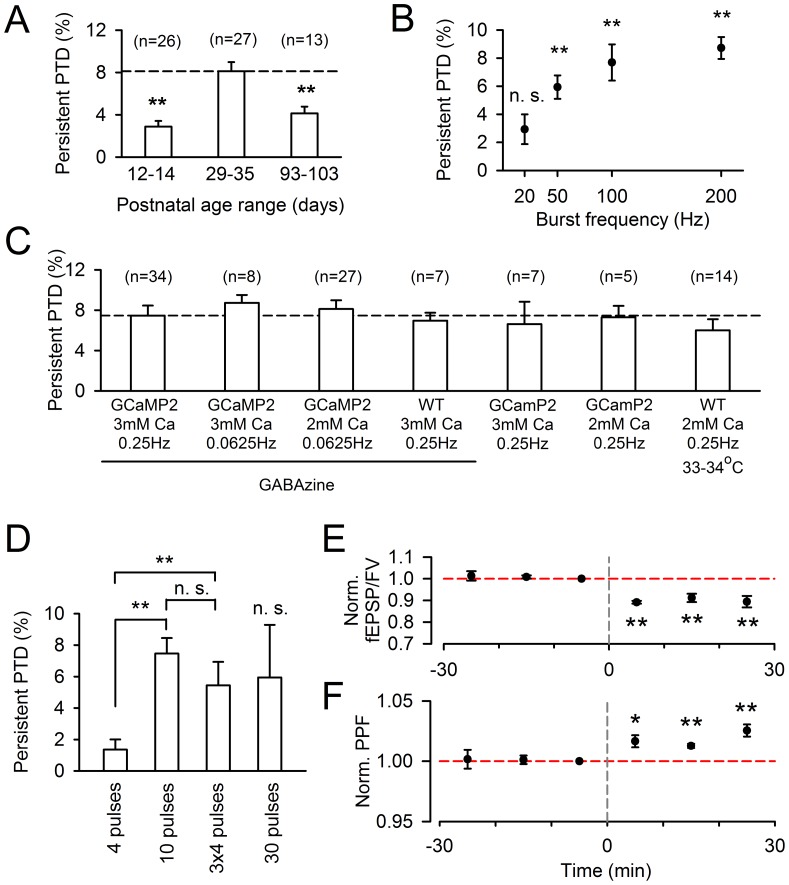
Conditions determining the observation of the persistent PTD. **A,** The persistent PTD is measured in slices from mice of postnatal ages 12–14 (average = 13.1±0.2, n = 26), 29–35 (average = 33.3±0.4, n = 27) and 93–103 (average = 98.4±0.9, n = 13). The persistent PTD is significant at all ages (p<0.02), but the amplitude is doubled for ages in the range of 29–35 (p<0.005). **B**, Persistent PTD amplitude plotted as a function of the frequency of 10-pulse bursts. 20, 50, 100 and 200 Hz are compared (n = 3, 5, 5 and 8 respectively). On average, the persistent PTD is significant for all frequencies above 50 Hz (p<0.004), but not for 20 Hz (p = 0.11). ***C***, Persistent PTD amplitude plotted for different conditions. The persistent PTD is not dependent on the synaptic transmission monitoring rate, as there is no significant difference between rates 0.25 Hz (n = 34) and 0.0625 Hz (n = 8; p = 0.32), indicating that the persistent PTD is not due to a fatigue of neurotransmission. Initial experiments were done in 3 mM extracellular [Ca^2+^]. The persistent PTD has identical amplitude in 2 mM extracellular [Ca^2+^] (n = 27; p = 0.62). The persistent PTD is identical in wild type animals (n = 7; p = 0.69). The persistent PTD is similar in absence of GABAzine (n = 7; p = 0.73) and at a near-physiological temperature (n = 14; p = 0.33). ***D***, Persistent PTD amplitude for different temporal patterns of 200 Hz trains. A 4 pulse train evokes a persistent PTD barely significant (p = 0.05; n = 35) and 6 times smaller than the persistent PTD evoked by a 10-pulse train (p = 3·10^−6^; n = 34). In comparison, the SSE evoked by a 4 pulse train is only 1.5 times smaller than the SSE evoked by a 10 pulse train (see Fig. 7A). Repeating the 4 pulse-trains 3 times with 100 ms intervals rescues the persistent PTD, which is not significantly different from the one induced by a 10 pulses train (p = 0.27; n = 11). A 30 pulse train led to high variability in the persistent PTD amplitude, which on average was not significant (not different from baseline; p = 0.11, n = 11). **E-F**, Following a ≥30 min stable baseline, the 10-pulse 200 Hz burst evokes a persistent PTD which lasts for at least 30 min. Averaged fEPSP/FV amplitude (**E**) and PPF (**C**) are normalized to the 10 min baseline prior burst stimulation. The change in PPF also appears to last for 30 min (p<0.01; two asterisks).

**Figure 6:**
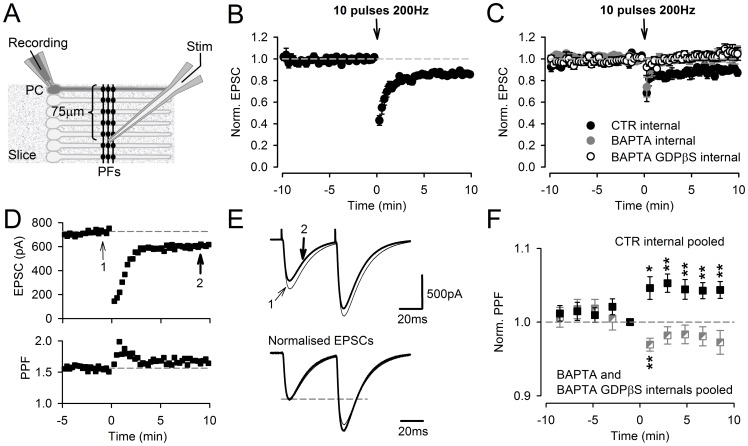
Patch-clamp recordings, the effect of postsynaptic calcium chelation. **A,** Schematic diagram showing the arrangement of the patch-clamped PC and the stimulating electrode. **B**, SSE and persistent PTD recorded in voltage-clamp mode following a burst of ten stimulations at 200 Hz applied in current-clamp mode. The plot shows the averaged EPSP amplitude normalized to the two minutes baseline preceding the burst (n = 9). Actual averaged EPSC amplitude is 500±63 pA. **C,** Abolition of the persistent PTD by the chelation of postsynaptic calcium. The burst stimulation was applied at least 40 min following the seal break for complete dialysis of the PC cytosol with intracellular media containing 10 mM EGTA (control; see Methods), 40 mM BAPTA or 40 mM BAPTA and 2 mM GDPβs (filled, grey or open symbols, n = 8, 7 or 9 respectively). Actual averaged EPSC amplitudes are 430±71 pA, 573±140 pA, 539±99 pA, respectively. **D,** Specimen voltage-clamp recording from a PC where a large EPSC was evoked (larger than the averaged EPSC amplitude which was 467±47 pA over the 17 control recording shown in B, C and F), showing a clear increase in PPF (bottom). **E**, Double-pulse evoked EPSCs from the same specimen recording, averaged over two minutes before the burst (thin trace; arrow 1 in **D**) and 8–10 min following the burst (thick trace; arrow 2 in **D**), are superimposed (top). The same EPSCs are scales relative to the amplitude of the EPSC evoked by the first pulse, illustrating the increase in the PPF plotted in **D** (bottom). **F,** Averaged change in PPF following the burst, for recordings obtained with the control internal (pooled data from recordings shown in **B** and those obtained after 40 min dialysis shown in **C**, n = 17; filled symbols) and recordings obtained with BAPTA-containing internals (pooled data, n = 16; half filled and grey symbols). One or two asterisks are for p<0.01 and p<0.005 respectively.

**Figure 7:**
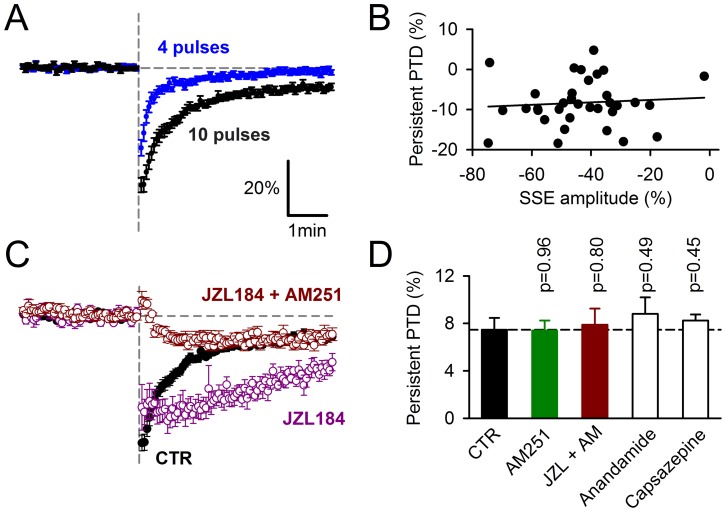
The persistent PTD is unaffected by manipulations of endocannabinoid signaling. *****A,***** Comparison of the averaged fEPSP/FV changes induced by 4 pulses (n = 35) or 10 pulses (n = 34) of PF stimulation at 200 Hz. **B,** Persistent PTD amplitude plotted against the SSE amplitude (10 pulses induction; n = 34). ***C,*** Prolongation of SSE in 1 µM JZL184. When 2 µM AM251 is added to JZL184, the remaining persistent PTD is undistinguishable from persistent PTD in AM251 alone (see Fig. 1H). ***D,*** Average persistent PTD amplitude in 2 µM AM251 (n = 13), 1 µM JZL184 and 2 µM AM251 (n = 12), 5 µM anandamide (n = 6), 10 µM capsazepine (n = 5).

**Figure 8:**
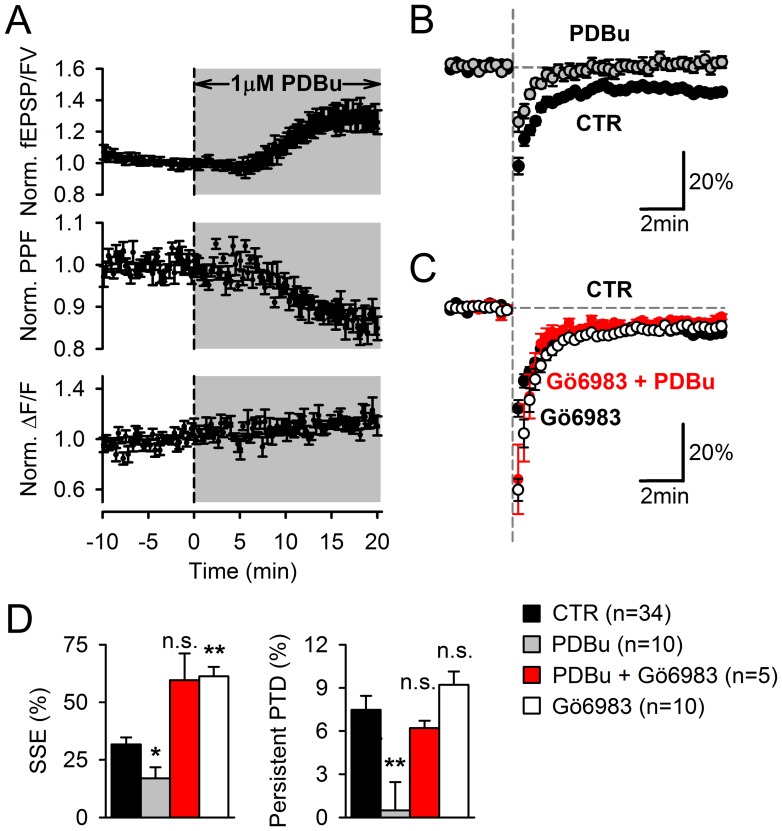
Modulators of DAG pathway affect the SSE and the persistent PTD differently. ***A,*** Application of 1 µM PDBu increases fEPSP/FV (top) and decreases PPF (middle) consistent with an increase in release probability. This increase in release probability does not appear to be mediated by an increase in presynaptic calcium signaling as indicated by the lack of effect of PDBu on ΔF/F signals arising from GCamP2 in PFs (bottom). ***B-C,*** fEPSP/FV is plotted. Vertical dashed grey line indicates stimulation with a 10-pulse burst at 200 Hz. In 1 µM PDBu, the SSE is reduced and the persistent PTD is abolished (n = 10) (***B***). When PDBu is applied in the presence of 2 µM Gö6983 (pre-incubated for 1 hour), a broad spectrum PKC blocker, the SSE is potentiated and the persistent PTD expression is rescued (n = 5) (***C***). Gö6983 alone has similar effects (n = 10) (***C***). ***D,*** Average effects of 1 µM PDBu, 1 µM PDBu +2 µM Gö6983 or 2 µM Gö6983 on the SSE or the persistent PTD amplitude. One or two asterisks are for p = 0.02 or p<0.01, respectively, relative to control conditions.

**Figure 9:**
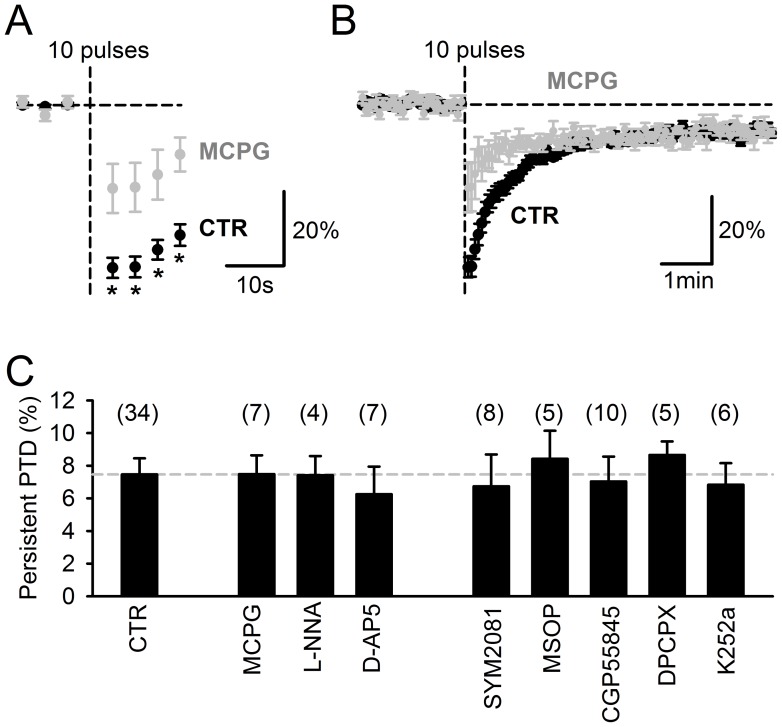
The persistent PTD is unaffected by the block of receptors reported to be required for postsynaptic LTD induction and involved in presynaptic modulation. *****A,***** The SSE is significantly decreased in 1 mM MCPG. The plot shows the average (n = 7) fEPSP/FV normalized to its averaged baseline value 2 min prior the 10-pulse burst. Asterisks are for p<0.02. ***B,*** Same plot on a longer time scale, showing that MCPG does not affect the persistent PTD, in contrast to its effect of the SSE. ***C,*** Summary bar chart showing the persistent PTD amplitude, 4–6 min following a 10-pulse 200 Hz burst. Blocker concentrations were (µM): MCPG (1000); L-NNA (100); D-AP5 (50); SYM2081 (10); MSOP (200); CGP55845 (5); DPCPX (5); K252a (2). In all conditions, the persistent PTD is significant (p<0.01) and not different from control (p>0.35).

**Figure 10:**
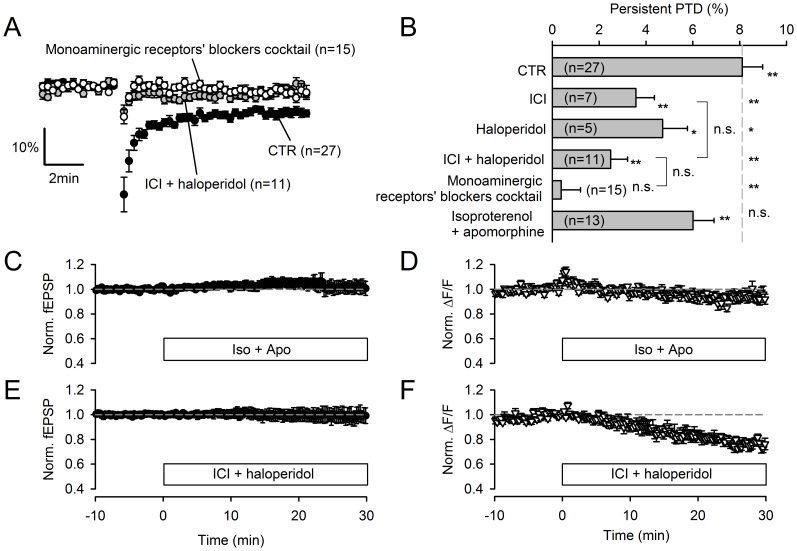
The persistent PTD is modulated by the monoaminergic system. **A,** The block of β-adrenergic and dopamine receptors inhibits the persistent PTD expression. In 10 µM ICI-118,551 and 10 µM haloperidol (grey symbols), the SSE and the persistent PTD are strongly reduced in amplitude. Additional block of other monoamine receptors by an additional 7 blockers (1 µM doxasosin, 1 µM asenapine, 10 µM thioperamide, 0.1 µM GR125487, 0.1 µM granisetron, 1 µM yohimbine, 10 µM scopolamine; open symbols) slightly further inhibits (p = 0.07) the persistent PTD (when averaging the 4–6 min time range). **B,** Average effects of pharmacological manipulations of the monoaminergic system. Both 10 µM ICI-118,551 and 10 µM haloperidol significantly inhibits the persistent PTD amplitude (p = 0.004 and p = 0.02 respectively). In contrast to the effect of blockers, the β-adrenergic and dopamine agonists isoproterenol (10 µM) and apomorphine (10 µM) do not significantly affect the persistent PTD amplitude (p = 0.10). One and two asterisks are for p<0.05 and p<0.01 respectively, compared to baseline or control persistent PTD amplitude. **C**, Following superfusion with agonists isoproterenol (10 µM) and apomorphine (10 µM), no detectable effect on the fEPSP is detected (n = 6). **D**, The two agonists induce a transient rise in the ΔF/F signal arising from GCaMP2 in the PFs, suggesting that β-adrenergic and dopamine receptors are not saturated in basal conditions. **E**, Superfusion with antagonists ICI-118,551 (10 µM) and haloperidol (10 µM) produced no significant effect on the fEPSP amplitude, ruling out the possibility that the block of the persistent PTD (**A**-**B**) is due to a direct effect of these antagonists on basal synaptic transmission. **F**, The two antagonists however induce a steady decrease in ΔF/F, indicating an action on PF calcium signaling.

The incubation medium contained (mM): NaCl 125, KCl 2.5, NaH_2_PO_4_ 1.25, NaHCO_3_ 25, NaNO_3_ 0.1, Na_2_SO_4_ 0.08, CaCl_2_ 2, MgCl_2_ 1, glucose 25, bubbled with 95% O_2_–5% CO_2_. The slicing medium contained (mM): KGluconate 130, KCl 15, EGTA 2, HEPES 20, Glucose 25, D-AP5 0.05 (to block NMDA receptors), pH adjusted to 7.4 with NaOH. This high potassium/EGTA (intracellular-mimicking) medium used solely during slicing at ∼4°C [20] enabled a better preservation of cells. Superfusion solution contained 10 µM GABAzine (to block GABA_A_ receptors) unless otherwise stated, Ca^2+^ concentration was 3 mM (Figs. 1–7), 2 mM (Fig. 8), 3 mM (Fig. 9), 2 mM (Fig. 10). The LTD amplitude was similar when using 2 or 3 mM extracellular Ca^2+^ (Fig. 3C), or in the absence of GABAzine (Fig. 3C). For patch-clamp experiments, normal intracellular (pipette) solution contained (mM): Kgluconate 110, KCl 2.4, CaCl_2_ 2, K_2.5_EGTA 10, HEPES 10, MgATP 4, NaGTP 0.5, Phosphocreatine-Na_2_ 5, pH adjusted to 7.3 with KOH. GABAzine, AM251, D-AP5, anandamide and capsazepine were purchased from Ascent Scientific; isoproterenol, apomorphine, K252a, Gö9683 and PDBu from Sigma-Aldrich; other drugs from Tocris. They were added directly in solutions (L-NNA, MCPG) or by dissolving stock solution made in water (GABAzine, TTX, NBQX, muscimol, D-AP5, SYM2081, MSOP, scopolamine, yohimbine, ICI-118551, granisetron, GR125487, isoproterenol, apomorphine) or dimethyl sulfoxide (AM251, JZL184, CGP55845, DPCPX, K252a, Gö6983, PDBu, anandamide, capsazepine, doxazosin, asenapine, haloperidol, thioperamide) whose final concentration never exceeded 0.1%. JZL184 was kindly provided by J. Z. Long and B. Cravatt. To facilitate the penetration of AM251, K252a, Gö6983 or JZL184, slices were pre-incubated with the drug for at least 2 hours before recording.

### Imaging

The excitation light was produced by a 468 nm LED (Cairn) with about 10 mW.mm^−2^ irradiance, and epifluorescence was collected through a x40 objective (Olympus; N.A. 0.8) and an emission filter at 529 nm (bandwidth 24 nm; Semrock) by an EM-CCD camera (iXon DU-897E; Andor). In order to monitor presynaptic residual calcium changes evoked by PF stimulation at 0.25 Hz or at 0.0625 Hz, the stimulator and the camera were driven externally by pCLAMP (Molecular Devices) to synchronize imaging and stimulation, and movies were captured at 0.5 Hz or 0.125 Hz respectively. Changes in GCaMP2 fluorescence (ΔF/F) were determined as follows. For each double-pulse stimulation, ΔF was obtained by subtracting the frame preceding the stimulation from the frame immediately following the stimulation. The slight decrease in fluorescence due to photobleaching was measured for molecular layer regions not affected by the stimulation, and taken into account to derive ΔF/F in the region of interest (ROI) covering the section of the activated PF beam. The boundary of the ROI was determined by adjusting the ΔF/F threshold until the ROI area equaled the total integrated ΔF/F (over the entire visible molecular layer) divided by the peak ΔF/F. In the text, ΔF/F refers to ΔF/F measurement over the ROI.

### Statistics

All experiments were preceded by a 10–40 min baseline recording. Experiments exhibiting a drift of FV, fEPSP or PPF of more than 1%/min during the 5 min prior induction were excluded from analysis. Data are presented as mean ± SEM. Significance was determined by a two-tailed t-test. Spearman Rank correlation was used to assess the significance of correlation between variables.

## Results

### A Single Burst of PF Firing Induces a Depression Extending Beyond the Short-term CB1-mediated Depression

Within sagittal slices of the cerebellar vermis, PF to PC transmission was monitored extracellularly by positioning the stimulation and recording electrodes in the transverse axis of PFs (Fig. 1A). Slices were superfused with 10 µM GABAzine to block GABA_A_ receptor-mediated transmission. The effect of stimulation near the surface of the slice was recorded about 75–100 µm vertically below the surface. The recorded field potentials displayed two negative peaks (N1 and N2) resolved from the stimulus artifact (Fig. 1B). N1 and N2 peak amplitudes were calculated after subtraction from recording traces obtained in TTX. The N1 peak was barely affected by NBQX (3.8±1.4%, n = 9; Fig. 1B–C), an efficient blocker of PF to PC fast synaptic transmission [21], and was hence assimilated to the fiber volley (FV), providing a readout of the action potential propagation along the stimulated PFs. In contrast, NBQX reduced the N2 peak by 98.2±0.3% (n = 9; Fig. 1B–C), indicating that the FV after potential previously reported (Ito & Kano, 1982) accounted for less than 2% of the N2 peak in our conditions. The N2 peak was thus assimilated to the field excitatory postsynaptic potential (fEPSP), providing a measure of PF to PC synaptic transmission. This type of recording enabled the monitoring of a population of unselected Purkinje cells from deep locations within the slice, where the tissue is better preserved. In addition, the monitoring of the FV provided a continuous assessment of the synaptic input preservation. The fEPSP gradually increased with stimulation intensity and exhibited paired-pulse facilitation (PPF; 1.25±0.03 for 50 ms apart stimuli, n = 34), as expected for PF inputs [22].

As shown in Fig. 1D, following a single burst of PF firing (10 pulses at 200 Hz), the fEPSP was transiently depressed (by ∼40%, 4–16 s following the burst), consistent with previous studies having monitored synaptic transmission by patch-clamp recording [16], [23]. However, unexpectedly, the fEPSP did not recover to baseline and remained significantly depressed after 10 min (Fig. 1D), revealing a persistent form of posttetanic depression (PTD). On average, 4–6 minutes following the burst, the fEPSP amplitude was 9.8±1.0% smaller than the baseline fEPSP (p = 3 10^−7^; n = 13; Fig. 1E) for recordings where the FV had not significantly changed over this time, demonstrating that the persistent PTD cannot be accounted for by damage of the input. On average, the FV decreased constantly by 0.43±0.13%·min^−1^ during recordings (n = 34; Fig. 1F). This slow decrease in the FV amplitude was identical during the 5 minutes preceding the induction and during the 5–10 min interval following the induction (p = 0.89; n = 34), indicating that the FV decrease was not promoted by the induction protocol. The effect of the FV variation on the fEPSP amplitude was examined during 10–40 min baseline recordings. The fEPSP amplitude exhibited a clear sub linear relationship with the FV amplitude (Fig. 1G). Thus, the normalization of the fEPSP over the FV provides a conservative estimate of the depression of synaptic transmission. To ensure that the depression measured is not a result of a decrease in the FV amplitude, fEPSP/FV is used throughout this study to quantify the persistent PTD (Fig. 1H). The depression reached a plateau after about 4 min (7.5±1% depression, n = 34, p = 10^−8^; Fig. 1H) and remained of constant amplitude thereafter (Fig. 1H–I). For consistency and accuracy, the depression was taken as the average between 4–6 min following the burst, unless otherwise stated. In contrast to the 1–2 min PTD previously reported [16], [17], [23] and named synaptically-evoked suppression of excitation (SSE; [24]), the persistent PTD was unaffected by the block of type 1 cannabinoid receptors CB1 (Fig. 1H–I). This indicates a clear distinction between the mechanism of the endocannabinoid-mediated PTD (SSE) and the persistent PTD reported here.

### Dependence on Glutamate Spillover

Stimulation in the molecular layer activates a beam of PFs whose synaptic boutons are in close proximity. This spatial pattern of synapse activation promotes glutamate spillover effects [17], [21], [25]. Here, the use of mice expressing the calcium indicator protein GCaMP2 in granule cells [19] enabled the imaging of action potential-evoked presynaptic calcium transients. According to the geometry of the PF to PC connection relative to the slice orientation (Fig. 1A), the section of excited PF beams was imaged. This image could be assimilated to a disk and provided a convenient measurement of the diameter of the activated PF beam (Fig. 2A). Consistently with previous finding that spatially sparse stimulation fails to induce SSE [17], [18], we found a correlation between the activated PF beam diameter (an index of stimulation intensity) and the SSE amplitude (p<0.001, n = 76; Fig. 2B). In contrast, the persistent PTD amplitude was identical over the same range of conditions (Fig. 2C).

The fEPSP evoked by train stimulation decayed more slowly than the fEPSP evoked by a single pulse (Fig. 2D), reflecting a longer extracellular glutamate transient due to local saturation of glutamate transporters [21], [25]. An index of the glutamate spillover effects, named spillover factor, is given by one tenth of the time-integrated fEPSP evoked by a 10-pulse train divided by the time-integrated fEPSP evoked by a single pulse. Although asynchronous release, known to be enhanced by train stimulation [26], may contribute to the calculated spillover factor, this contribution is expected to be negligible compared to the effect of glutamate spillover which prolongs the AMPA-mediated response [21] and evokes a slow mGluR1-mediated response [17]. Predictably [17], we found a high correlation between the SSE amplitude and the spillover factor (Fig. 2E). In striking contrast, the persistent PTD amplitude did not correlate with the spillover factor (Fig. 2F). We conclude that the persistent PTD induction mechanism differs from that of the SSE regarding its dependency on the spatial pattern of the input and glutamate spillover.

### The Persistent PTD and Presynaptic Calcium Signaling

The effect of the burst stimulation on the action potential-evoked presynaptic calcium change was monitored as the change in GCaMP2 fluorescence (ΔF/F; see Methods). The low signal-to-noise ratio of these measurements produced an apparent large variability of the ΔF/F (Fig. 3A) compared to the corresponding monitored fEPSP/FV (Fig.3B). Despite this variability, the distribution of the ΔF/F change measured at the 4–6 min time point was not significantly different from zero (mean  = 1.4±1.0%, n = 107, p = 0.14; Fig. 3C), in contrast to the distribution of the change in fEPSP/FV amplitude (−7.51±0.41%, n = 107; Fig. 3D), suggesting that the persistent PTD is not caused by a change in presynaptic calcium signaling. In contrast to the endocannabinoid-mediated SSE, the early development of the persistent PTD revealed in AM251 (Fig. 3B; see below) does not exhibit any detectable change in ΔF/F (Fig. 3A). Finally, the persistent PTD amplitude plotted against the change in ΔF/F showed no significant correlation (n = 107; Fig. 3E). Together, these results indicate that, unlike the SSE and the presynaptic LTD unmasked by the block of PKA [14], the persistent PTD is not associated with any detectable change in presynaptic residual calcium signaling, as probed by GCaMP2. This does not rule out possible roles of presynaptic calcium signaling below our detection threshold.

### Changes in Paired-pulse Facilitation and in Coefficient of Variation

In contrast to the postsynaptic LTD [8], [11], the persistent PTD was associated with an increase in paired-pulse facilitation (PPF), a short-term plasticity whose amplitude correlates with release probability [22]. This change was small (2.5±0.4%, n = 27), but highly significant (p<0.004; Fig. 4A). The apparent small size of this change is not surprising given the small size of the persistent PTD amplitude. In fact, the plot in Fig. 4B shows that the 2.5% change is consistent with what would be expected if depression of synaptic transmission was entirely caused by a presynaptic decrease in release probability. Given the established presynaptic expression of the SSE [16], [17] and the correlation between the PPF and the release probability at the PF to PC synapse [22], this plot shows that the change in PPF associated with the persistent PTD is within the range of change in PPF associated with change in neurotransmitter release. The coefficient of variation of the postsynaptic responses was significantly increased following the induction (p<0.05, n = 27; Fig. 4C), further supporting a decrease in presynaptic release probability [27].

### Basic Requirements for the Persistent PTD Induction

A series of experiments were conducted to further describe the conditions required to evoke the persistent PTD. The persistent PTD exhibited a developmental dependence: it was maximal at pre-adolescent age (P29–35) and half maximal for other ages tested (Fig. 5A). The persistent PTD amplitude increased with firing rate and could start being detected for 10 pulses at 50 Hz (Fig. 5B). However, increasing the number of pulses in the train produced a biphasic effect with 10 pulses in a train appearing as the optimal condition to observe the persistent PTD (Fig. 5D). A protocol of three 4 pulse-trains interleaved by 100 ms produced a persistent PTD of similar amplitude as the one produced by a 10-pulse train (Fig. 5D). The persistent PTD amplitude did not appear to depend on extracellular calcium concentration (p = 0.79; range 2–3 mM tested; Fig. 5C). It was similar in GCaMP2 and wild type mice (p = 0.69; Fig. 5C). It was similarly observed when GABAzine was omitted (p = 0.73; Fig. 5C). It was not significantly different when recording at near physiological temperature (p = 0.33; Fig. 5C). Importantly, it did not depend on the stimulation monitoring rate (p = 0.32; Fig. 5C), ruling out an effect of synaptic transmission fatigue. Furthermore, the range of used monitoring rates (0.0625–0.25 Hz) is far below the estimated average granule cell firing rate in vivo (∼3 Hz; [28]). Finally, the depression and increased PPF remained for at least 30 min (n = 5, p<0.01; Fig. 5E–F), to suggest that this persistent PTD is long-term.

### Requirement for Postsynaptic Calcium Signaling

When the excitatory postsynaptic current (EPSC) evoked by PF stimulation was monitored from PCs (Fig. 6A), which implies the dialysis of our standard intracellular medium (see Methods) into the PC cytoplasm, the persistent PTD was still observed (Fig. 6B), with an average amplitude of 14.9±1.7% (n = 9, p = 3·10^−5^). Forty minutes dialysis of the recorded PC did not have any significant effect on the persistent PTD amplitude (12.2±2.7%, n = 8; p = 0.42; Fig. 6C). When the recorded PC had been dialyzed for 30–40 min with an intracellular medium containing either BAPTA (in order to chelate postsynaptic calcium; n = 7) or BAPTA and GDPβS (in order to disrupt G-protein signaling; n = 9), the persistent PTD was consistently abolished (the EPSC recovered to values not significantly different from baseline 4 min following the induction, p>0.15; Fig. 6C, grey and open symbols). These data strongly suggest that the induction of the persistent PTD requires postsynaptic signaling. The effect of BAPTA alone argues for a role of postsynaptic calcium signaling. In support of the data depicted in Fig. 4, the persistent PTD monitored by patch-clamp experiment exhibited a change in PPF (Fig. 6D–F).

### Unlike the SSE, the Persistent PTD is not Endocannabinoid Mediated

To reconcile the evidence that the persistent PTD is expressed presynaptically and yet requires postsynaptic signaling for its induction, we postulate the involvement of a retrograde messenger. The most established retrograde messenger at the PF to PC synapse is the endocannabinoid 2-arachidonoyl glycerol (2-AG) [29]. The suppression of the SSE when using the BAPTA/GDP-βS internal, indicates the suppression of 2-AG release from the patched cell (Fig. 6C) [16]. The abolition of the persistent PTD in these conditions could be explained if the persistent PTD was mediated by 2-AG. AM251, a blocker of type 1 cannabinoid (CB1) receptors left the persistent PTD unaltered, while abolishing the SSE (Fig. 1H), ruling out the requirement of CB1 receptor activation for persistent PTD induction or expression. However, this does not rule out the involvement of 2-AG which could act on other targets [30]. A 4-pulse burst evoked an SSE slightly reduced in amplitude (28.6±2.9%, n = 35) compared to that evoked by the 10-pulse burst (43.4±2.7%, n = 34; Fig. 7A). This 34% reduction was presumably due to a reduction in the amount of 2-AG release. The persistent PTD evoked by a 4-pulse burst was barely detectable (1.3±0.7%, n = 35, p = 0.05). Two hypotheses can be proposed to interpret this observation: either the persistent PTD is not mediated by 2-AG, or 2-AG is less effective to induce the persistent PTD than it is to induce the SSE. The former hypothesis is supported by the lack of correlation between the SSE and the persistent PTD amplitudes (Fig. 7B). We tested the second hypothesis by enhancing the amount of 2-AG release by the block of 2-AG degradation by 1 µM JZL184, a potent specific inhibitor of the monoacyl glycerol lipase [31]. If 2-AG were little effective to induce the persistent PTD, increasing its amount would be expected to increase the persistent PTD amplitude. In JZL184, the SSE was dramatically prolonged (Fig. 7C), indicating increased or prolonged levels of 2-AG. In contrast, the superimposed persistent PTD, revealed with the block of CB1 receptors with AM251, was unaffected (p = 0.8, n = 12; Fig. 7C–D). Together these results support the conclusion that 2-AG does not mediate the persistent PTD.

Anandamide has been recently proposed to mediate plasticity via the activation of vanilloid TRPV1 receptors [32], [33], [34], [35]. We tested this possibility by either incubating slices with 5 µM anandamide (to saturate anandamide binding sites), or blocking TRPV1 by 10 µM capsazepin. None of these conditions affected the persistent PTD amplitude (Fig. 7D).

### Presynaptic Mechanism of Persistent PTD Expression

Since the persistent PTD is associated with a change in PPF consistent with a decrease in glutamate release probability (Fig. 4), we explored the presynaptic mechanism that might be involved. Our presynaptic calcium recordings did not reveal any significant change in presynaptic calcium signaling once the SSE had returned to baseline (Fig. 3). A role for presynaptic diacylglycerol (DAG) in the modulation of neurotransmitter release has been proposed based on the potentiating effect of phorbol esters, functional analogs of DAG that bind to C1 domains found notably in Protein Kinase C (PKC) and Munc-13 [36], [37], [38]. Twenty minutes superfusion with 1 µM phorbol 12,13-dibutyrate (PDBu) potentiated the fEPSP/FV by 29.8±6.8% (p = 0.007) while reducing the PPF by 12.3±1.6% (p = 0.0006, n = 6; Fig. 8A), indicating an increase in release probability. This effect is consistent with the presynaptic action of PDBu previously reported [36], [38]. The lack of change in presynaptic ΔF/F following PDBu application (Fig. 8A), indicates that its effect is not mediated by a modulation of presynaptic calcium signaling.

Following PDBu application, the SSE was reduced by 54% compared to control (to 17±4.8%, n = 8, p = 0.02), and strikingly, the persistent PTD was abolished (not significantly different from zero, n = 10, p = 0.83; Fig. 8B,D). This effect was unlikely to be due to an increase of basal glutamate release and AMPA receptor saturation because these experiments were done in 2 mM Ca^2+^ and enhanced release in 3 mM Ca^2+^ had no detectable effect on the persistent PTD (Fig. 5C). To test whether PDBu effects were PKC-mediated [39], slices were incubated with the PKC blocker Gö6983 (at 2 µM for at least 2 h). The SSE was potentiated by 93% relative to control (61.3±4.1%, n = 10, p = 10^−5^) and the persistent PTD was rescued (Fig. 8C,D). Together with the lack of change in ΔF/F associated to the persistent PTD expression, these data suggest that PKC activation by presynaptic DAG prevents the expression of the persistent PTD.

### Mechanism of Induction

In order to test whether the postsynaptic LTD might partly contribute to the persistent PTD we report, we examined the effect of blocking signaling pathways reported to mediate postsynaptic LTD [5], [8], [40], [41], [42]. The blockade of type 1 metabotropic glutamate receptors (mGluR1) with 1 mM MCPG significantly reduced the SSE amplitude (p<0.02 for up to 16 s following induction; Fig. 9A), as expected [43], but left persistent PTD unaffected (Fig. 9B; p = 0.99, n = 7). Blockers of nitric oxide synthase (100 µM L-NNA) or NMDA receptors (50 µM D-AP5) also failed to affect the persistent PTD (p = 0.97 and 0.55 respectively; Fig. 9C). We conclude that the signaling pathways involved in the persistent PTD induction differ from those reported to mediate postsynaptic LTD induction.

It has recently been suggested that part of the short-term depression of PF synapses evoked by depolarization of PCs is mediated by glutamate binding to presynaptic kainate receptors, rather than an endocannabinoid-mediated mechanism [44]. However, in contrast to the short-term depression reported by [44], the persistent PTD herein was not significantly affected by 10 µM SYM2081, a desensitizing agonist of kainate receptors (p = 0.75; Fig. 9C). NMDA and mGluR4 receptors have also been reported to be expressed at PFs [5], [45]. Their respective blockers D-AP5 (50 µM) and MSOP (200 µM) did not significantly affect the persistent PTD (p>0.55; Fig. 9C), arguing against glutamate as the retrograde signal mediating the persistent PTD. GABA_B_, type 1 adenosine (A1) and TrkB receptors have been shown to be expressed on PFs [46], [47], [48]. However, their blockade (with CGP55845, DPCPX and K252a respectively) did not significantly affect the persistent PTD (p = 0.51, p = 0.37, p = 0.71 respectively; Fig. 9C). The lack of change in presynaptic calcium signaling (Fig. 3) further rules out presynaptic mGluR4, GABA_B_ or A1 receptors, whose known effects on glutamate release are calcium-mediated.

### Modulation by the Noradrenergic and Dopaminergic Systems

The role of monoamine innervations to the cerebellar cortex is poorly understood [49]. Noradrenergic afferents are dense in the molecular layer [50] and noradrenalin has been proposed to modulate GC to PC transmission [51]. Dopamine receptors are expressed throughout the molecular layer [52]. We found that the block of β-adrenergic receptors by 10 µM ICI-118,551 or dopamine receptors by 10 µM haloperidol significantly reduced the amplitude of the persistent PTD by about 50% (p = 0.001, n = 7 and p = 0.03, n = 5 respectively; Fig. 10B). The combination of the two blockers inhibited the persistent PTD amplitude by 69% (p = 2·10^−5^, n = 11; Fig. 10A,B). The addition of 7 blockers to span most of the monoamine receptors further reduced the persistent PTD amplitude, albeit non-significantly compared to the effect of combined ICI-118,551 and haloperidol (p = 0.07, n = 15; Fig. 10A,B). The mediation of the persistent PTD by noradrenalin and dopamine was ruled out: first, the application of the β-adrenergic receptor and dopamine receptor agonists isoproterenol (10 µM) and apomorphine (10 µM) did not induce any detectable reduction of the fEPSP (Fig. 10C); second, isoproterenol and apomorphine did not prevent the persistent PTD induction as would have been expected by occlusion (p = 0.1, n = 13; Fig. 10B). We conclude that the persistent PTD induction is permitted by tonic activation of β-adrenergic and dopamine receptors, which may be borne by PFs, as suggested by the transient effect of isoproterenol and apomorphine and the slowly developing inhibitory effect of monoamine receptor blockers on ΔF/F (Fig. 10E,F). Since the persistent PTD does not depend on the stimulation intensity (Fig. 2B) or the monitoring stimulation rate (Fig. 5C), it is unlikely that a tonic noradrenalin and/or dopamine concentration rise results from the recruitment of monoaminergic fibers by our PF stimulation.

## Discussion

We have characterized a novel form of synaptic depression at the GC to PC synapse. The depression is evoked by a single train of 10 PF action potentials (or 3 successive trains of 4 PF action potentials; see Fig. 5D) at high frequency, a protocol similar to the one known to evoke the previously reported short-term synaptically-evoked suppression of excitation (SSE) [16], [17], [23], [24]. This depression lasts for 20 minutes at least. Its total duration remains unclear, its small amplitude complicating examination at later time points. Rather than trying to modify our stimulation protocol to evoke plasticity of enhanced amplitude (for example by repetitive burst stimulations), we limited our investigation to the effect of a the single burst protocol, the physiological relevance of which being strongly supported by the responses to sensory inputs *in vivo* [15]. Thus we refer to the novel plasticity we report as a persistent posttetanic depression (persistent PTD).

The persistent PTD clearly differs from the SSE: the block of CB1 receptors has no effect on the persistent PTD while it abolishes the SSE (Fig. 1H; [16], [17]); the persistent PTD lasts at least 20 min while the SSE typically recovers to baseline within 2 min (Fig. 5E; [16], [17]); the persistent PTD is not associated with a change in presynaptic calcium signaling while the SSE is (Fig. 3A; [16]); the persistent PTD does not depend on input density and glutamate spillover effects as the SSE does (Fig. 2; [21], [53]). In contrast to the classic postsynaptic LTD [5], [41], [54], [55], the persistent PTD is associated with an increase in the coefficient of variation and the PPF of synaptic responses (Fig. 4 and 6D–F) and is unaffected by blockers of mGluR1, NMDA receptors, NOS or PKC (Figs. 8 and 9). Recently, it has been reported that CB1 receptors are required to observe the postsynaptic LTD [7] and a new form of presynaptic LTD unmasked by the pharmacological block of the presynaptic LTP [14]. In contrast, the persistent PTD is independent of CB1 receptor signalling. The persistent PTD lasts longer than other forms of short-term plasticity previously described at this synapses [56], [57], and its protocol of induction is much simpler than those used to evoke long-term plasticity.

The changes in PPF and coefficient of variation (Fig. 4 and 6D–F) suggest that that the persistent PTD is expressed presynaptically, as a decrease in neurotransmitter release probability. However, the persistent PTD could not be induced when postsynaptic calcium was chelated (Fig. 6C). Together, these observations are reminiscent of the retrograde endocannabinoid system [24]. The possibility that the endocannabinoid 2-AG [29], mediates the persistent PTD can be ruled out since manipulations of release or degradation of 2-AG strongly altered the SSE amplitude, while leaving the persistent PTD amplitude unaffected (Fig. 7). The other endocannabinoid anandamide, recently reported to mediate forms of plasticity through activation of TRPV1 receptors [32], [33], [34], [35], does not appear to be involved since the persistent PTD was neither affected by occlusion with 10 µM anandamide nor by TRPV1 blockade (Fig. 7D). Glutamate has been proposed to mediate two types of presynaptic short-term plasticity mechanisms induced by postsynaptic depolarization [44], [58]. However, the blockade of signaling mediated by glutamate receptors expressed by PFs (kainate, mGluR4 or NMDA receptors) did not affect the persistent PTD (Fig. 9C). Furthermore, the persistent PTD amplitude did not correlate with the amount of glutamate spillover (Fig. 2F), as would have been expected if it was mediated by glutamate signaling to the presynaptic site [44]. Finally, pharmacological investigation rules out the most obvious other candidates GABA, adenosine, ATP, BDNF and NO (Figs. 5C and 9C). This screen of most of the putative extracellular signaling molecules reported to be involved at the PF synapse has not identified any retrograde messenger that mediated the persistent PTD. We conclude that either the nature of the retrograde messenger is novel and its quest may take years of investigation, or despite the evidence for a change in release probability (Fig. 4 and 6D-F) the persistent PTD is expressed post-synaptically. Indeed, our arguments supporting a change in release probability remain indirect. The use of low-affinity AMPA receptor antagonists [59] may be a fruitful strategy to resolve this issue in future studies.

The inhibition of the persistent PTD by the functional analog of DAG, PDBu, is reversed by the PKC inhibitor Gö6983, suggesting that the persistent PTD block is PKC-mediated. PKC-mediated phosphorylation of N-type calcium channels has been shown to hamper calcium channel modulation by the G protein βγ subunit [60]. This mechanism may account for the strong modulation of the SSE but is unlikely to account for the modulation of the persistent PTD, which does not appear to be mediated by a change in presynaptic calcium signaling (Fig. 3). Other PKC targets or proteins possessing the DAG binding domain C1, like Munc-13 [61], which is capable of modulating neurotransmitter release [37], [62], but whose role is poorly understood, may mediate the persistent PTD blockade by PDBu.

It is surprising that this long lasting effect of a single PF burst has not been reported earlier. This may be due to the following reasons. (i) The small amplitude of the persistent PTD may have hampered its detection, as it requires a fairly long and stable baseline recording prior to the burst and control experiments to check that the small change is not due to a loss of inputs (we checked that by monitoring the FV). (ii) The persistent PTD expression depends on the internal medium used when the EPSC is recorded in whole-cell mode. We have shown the sharp dependency on postsynaptic calcium chelation, but there may be other parameters in the internal we used which are critical. (iii) Most investigations of the GC to PC plasticity have used juvenile or adult animals while the optimal age to observe the persistent PTD is around P32 (Fig. 5A). (iv) The monoamine modulation we reveal by blocking β-adrenergic and dopamine receptors suggests that tonic noradrenalin and dopamine release occurs within our acute slices. This tonic release may vary depending on the procedure used to obtain acute slices.

What is the physiological relevance of the persistent PTD? The correlation between the amplitude of a plasticity mechanism and its physiological relevance is arbitrary. Mechanisms producing large amplitude plasticity may generate instability of neuronal networks. The plasticity we report is induced by a protocol far simpler and more physiological (single short high frequency burst) than the protocols used previously (hundreds of repeated stimulations at low frequency). The lack of dependence on the density of input and glutamate spillover promoted by the experimental direct stimulation of PFs (Fig. 2) further supports the physiological relevance of the persistent PTD.

The recent finding of a role of postsynaptic LTP in motor learning [63] has challenged the classic view that the postsynaptic LTD is the main synaptic plasticity underlying cerebellar motor learning [64]. Similarly to the postsynaptic LTP, the non-associative property of the persistent PTD does not rule out its role in motor learning. Alternatively, its role may be to dynamically reset synapses to low gain, consistent with the suggestion that the majority of PF to PC synapses are silent [1], [65], [66].
